# An observational study of self-monitoring in ad hoc health care teams

**DOI:** 10.1186/s12909-020-02115-3

**Published:** 2020-06-23

**Authors:** Stefanie C. Hautz, Daniel L. Oberholzer, Julia Freytag, Aristomenis Exadaktylos, Juliane E. Kämmer, Thomas C. Sauter, Wolf E. Hautz

**Affiliations:** 1 Department of Emergency Medicine, Inselspital University Hospital Bern, Freiburgstrasse 16c, 3010 Bern, Switzerland; 2 Simulated Patient Program, Office of the Vice Dean for Teaching and Learning, Charité – Universitätsmedizin Berlin, corporate member of Freie Universität Berlin, Humboldt-Universität zu Berlin, and Berlin Institute of Health, Charitéplatz 1, 10117 Berlin, Germany; 3 Max Planck Institute for Human Development, Center for Adaptive Rationality (ARC), Lentzeallee 94, 14195 Berlin, Germany; 4 Lernzentrum, Charité – Universitätsmedizin Berlin, Charitéplatz 1, 10117 Berlin, Germany

**Keywords:** Debriefing, Self-monitoring, Ad hoc teams, Resuscitation, Emergency medicine, Postgraduate education, Interprofessional education

## Abstract

**Background:**

Working in ad hoc teams in a health care environment is frequent but a challenging and complex undertaking. One way for teams to refine their teamwork could be through post-resuscitation reflection and debriefing. However, this would require that teams have insight into the quality of their teamwork. This study investigates (1) the accuracy of the self-monitoring of ad hoc resuscitation teams and their leaders relative to external observations of their teamwork and (2) the relationship of team self-monitoring and external observations to objective performance measures.

**Methods:**

We conducted a quantitative observational study of real-world ad hoc interprofessional teams responding to a simulated cardiac arrest in an emergency room. Teams consisting of residents, consultants, and nurses were confronted with an unexpected, simulated, standardized cardiac arrest situation. Their teamwork was videotaped to allow for subsequent external evaluation on the team emergency assessment measure (TEAM) checklist. In addition, objective performance measures such as time to defibrillation were collected. All participants completed a demographic questionnaire prior to the simulation and a questionnaire tapping their perceptions of teamwork directly after it.

**Results:**

22 teams consisting of 115 health care professionals showed highly variable performance. All performance measures intercorrelated significantly, with the exception of team leaders’ evaluations of teamwork, which were not related to any other measures. Neither team size nor cumulative experience were correlated with any measures, but teams led by younger leaders performed better than those led by older ones.

**Conclusion:**

Team members seem to have better insight into their team’s teamwork than team leaders. As a practical consequence, the decision to debrief and the debriefing itself after a resuscitation should be informed by team members, not just leaders.

## Background

Health care is an inherently social and interdisciplinary endeavour [1, 2]. Take, for example, emergency departments, where teams of physicians, nurses, and other health care professionals are routinely involved in diagnosing and treating patients [1, 3–5]. Collaboration is particularly common in high-urgency situations such as trauma calls or resuscitations that are typically handled by ad hoc teams (also known as health care action teams or interdisciplinary action teams): interdisciplinary, interprofessional groups of specialized individuals who work together in a highly dynamic, complex situation and under time pressure to accomplish critical tasks [6].

Especially when time is of the essence, the performance of such teams critically depends on the quality of their teamwork [7, 8], a term that summarizes “*human factors and non-technical skills (leadership, [collaboration], situation awareness and decision making*)” [9]. For example, Tiel and colleagues demonstrated that patient mortality is lower when trauma teams are trained in teamwork, including leadership skills [10]. One such key skill is coordination [11] or, as Marks et al. put it, “orchestrating the sequence and the timing of interdependent actions” [12]. Teamwork in emergencies furthermore should be contextually adaptive and account for team member experience and patient acuity [13].

Obviously, developing such teamwork requires training and experience of team members. Plenty of publications describe simulations to address this need (e.g., [14–19]). A few common training aims, often referred to as crew (or crisis) resource management principles (CRM), are widely adopted [20]. This conception implicitly assumes that training CRM in one ad-hoc team transfers to other ad-hoc teams, in which trainees may find themselves in the next day. However, it remains unclear if that is indeed the case and whether and how the skills acquired in such simulations translate to clinical practice [21, 22]. Fortunately, clinical practice itself also provides many opportunities for real-life ad hoc teams to (further) refine their teamwork.

Several tools have been developed to guide feedback to resuscitation teams by scaffolding outside observations (e.g., [9, 23–26]). Post-resuscitation debriefing, for example, is recommended by resuscitation guidelines internationally [27, 28] to facilitate reflection upon teamwork as it has been associated with important patient outcomes such as return of spontaneous circulation and shorter no-flow time in cardiopulmonary resuscitation. The use of these tools requires, however, the presence of a trained observer, which poses both logistical and ethical challenges [29]. Because ad hoc teams, particularly in emergency care, can be needed at any time of any day, it is rarely feasible to ensure an observer’s availability. In addition, it may be ethically unacceptable to limit the outside expert’s role to observation if the team’s performance is less than optimal. Yet, prompt intervention would disturb both the team and the collection of information for later debriefing.

One potential way to inform debriefing that circumvents the logistical and ethical challenges is self-reflection of the team. Several frameworks for post-resuscitation debriefing exploit that possibility in that they ask the team to reflect on “what went well” and “what could have gone better”, for example [30]. Almost all emergency rooms (ERs) establish the role of a physician team leader in their resuscitation teams. Because most post-resuscitation debriefing frameworks call upon the physician team leader or the charge nurse to decide whether a debrief is required at all (e.g. [30]), the accuracy of their self-evaluation is of critical importance.

But are teams and their leaders accurate judges of their own teamwork? The literature on self-monitoring suggests so [31–33]: Although it has repeatedly been demonstrated that individuals’ *self-assessments* are low in accuracy [34, 35], people are much better able to *self-monitor* their current performance [31–33]. The key conceptual difference between self-assessment and self-monitoring is the timing of introspection: self-assessment refers to a summative overall self-evaluation detached from a single event (e.g., “How good a team player am I?”), whereas self-monitoring is a moment-to-moment assessment of one’s performance in a given situation (e.g., “How is our teamwork in this particular case?”) [35]. Self-monitoring is what prompts people to “slow down when they should” [33] or to look up a word when they are unsure of its meaning [35]. Self-reflection of team members before debriefing within a team directly after attending to a patient is conceptually closer to self-monitoring than to self-assessment, because the reflection refers to a single event in close temporal proximity, not an overall and aggregate judgement.

Based on this conception of self-monitoring, we hypothesize that the ability of both teams and their physician team leaders to self-monitor their teamwork directly after attending to a patient will be comparable to that of an external observer. Such an ability would justify relying on team members’ self-monitoring to inform debriefing in real-life teams, thus easing and facilitating training on the job.

Furthermore, we investigated whether the ratings of teamwork provided by the team, their leader and external observers are related to objective measures of performance that directly affect patient outcomes [36]. It is arguably only clinically meaningful and defensible to assess how well teams and their physician team leaders self-monitor their teamwork relative to an external observer if either the team’s self-monitoring judgements or the external observations are related to direct patient outcomes or measures of proven importance.

This quantitative observational study therefore addressed the following research questions:

1. How accurately do ad hoc resuscitation teams and their leaders monitor their teamwork relative to external observers’ rating of that teamwork?
2. How do teams’ self-monitoring judgements and external observations of their teamwork relate to objective performance measures?

## Methods

We conducted an observational study of real-world interprofessional ad hoc teams responding to a simulated cardiac arrest in the emergency room. Teams consisting of residents, consultants, and nurses were confronted with an unexpected, simulated, standardized cardiac arrest situation. Their performance was videotaped to allow for subsequent external evaluation.

### Participants, teams, and setting

The study was conducted in a single university-affiliated level-one emergency room attending to more than 50.000 patients annually [37]. All medical staff in this emergency room are required to attend an interprofessional training day once annually. Staff were assigned to one of 10 training days in their work schedule; participation in these trainings is mandatory. On each training day, participants were randomly assigned to one of four teams. Randomization was stratified by profession, so that each team included at least one physician and two nurses. Depending on training day attendance, some teams were larger than others, including up to three physicians and up to four nurses, reflecting team variability in clinical practice. The most senior physician in each team was appointed *physician team leader* upon team formation, reflecting clinical practice in the emergency room under investigation. We did not provide further instructions to physician team leaders because all participating physicians regularly fulfil this role in clinical practice, and we did not expect any differences between that role in the simulation and clinical practice.

### Simulation and scenario

The 2019 interprofessional training day was designed as a rotation through different skill stations, where participants trained techniques such as intraosseous access, patient positioning, and paediatric advanced life support. Further, all teams rotated to a simulated shift handover, which was unexpectedly interrupted by a resuscitation call. Teams attended to this call as they would in reality; the simulation was designed as an in situ simulation and took place in the actual resuscitation bay of the emergency room.

In this resuscitation bay, teams were confronted with an elderly male patient on a stretcher (represented by a Leardal SimMan Essential patient simulator) under ongoing cardiopulmonary resuscitation (CPR) by paramedics. The paramedics reported to have initially encountered a conscious but helpless elderly patient in his flat. There, the patient was found lying on the floor after tripping over the sill of his balcony door 10 h previously when coming back inside from his balcony. He had fallen from body height and had been unable to alert help for an extended period up until a neighbour heard his calls for help. The paramedics found a hemodynamically stable, cold, and conscious but disoriented patient. They established a venous access and transferred the patient to the hospital uneventfully. Upon transfer from the ambulance onto the hospital’s stretcher just minutes prior, the patient had gone into cardiac arrest. The paramedics started CPR and alerted the ER staff.

The paramedics repeatedly informed the attending ER team that the patient’s flat was cold due to the balcony door being open and that the patient was cold as well. Paramedics had just initiated basic life support. Neither medication nor defibrillation had yet been provided. The paramedics then handed over resuscitation to the ERs team but remained available for further questions while they reorganized their material.

The patient simulator was programmed to exhibit a ventricular fibrillation and not to respond to resuscitative measures for the next 15 min. The team worked in its familiar emergency room environment with its usual equipment. Whenever a team member providing chest compressions was replaced, a study aid quietly informed them that their hands felt very cold due to the patient’s cold chest. This was necessary because the simulator used could not be programmed to feel cold. Whenever a team decided to take the patient’s temperature, the thermometer read 27.3 °C independent of the location of temperature measurement. All phone calls made by the team were answered by the simulation instructors, who responded with a standard response indicating that they would be available within a few minutes.

The simulation was wound up as soon as teams called for extracorporeal circulation (ECC) due to hypothermic arrest or ended by the instructor if teams had clearly made a different diagnosis of why the patient was in arrest. The whole scenario was intended to last less than 15 min. It was followed by a structured, instructor-led debriefing that included reviewing a video recording of the simulation. The debriefing was led by a trained and experienced simulation instructor and revolved around teamwork, team leadership and non-technical skills.

### Ethics

The ethics committee of the Canton of Bern deemed the study to be exempt from full ethical review (Req-2017-00968) because it did not involve patients. All participants provided written informed consent for their data to be used in the study. As an incentive, all participants were entered in a lottery for the chance to win one of two tablet computers.

### Measurement instruments

#### Participant questionnaires

All participants in the study independently answered two custom questionnaires. The first, which was administered prior to the simulation scenario, collected demographic information, data on participants’ professional experience, and information on their degree of acquaintance with the other team members. The data were collected as numbers (age, experience), through checkboxes (gender), or on 5-point Likert scales (acquaintance). The second questionnaire was completed individually directly after the simulation scenario and before debriefing. It assessed participants’ familiarity with the task (“Please indicate how familiar you are with cases like the one you just encountered.”) and their self-monitoring of their team’s teamwork (“Please indicate your confidence in the quality of your team’s teamwork.”) on 5-point Likert scales. It is generally assumed that the resulting confidence scores reflect the metacognitive feeling of fluency (see e.g., [38]). Previous research has shown a close relation of such confidence scores to other indicators of fluency, such as response time on diagnostic tasks [39] and the likelihood to change an initial answer in multiple choice tests [40].

#### Video recordings and choice of rating tool

All simulations were video recorded and recordings were evaluated by independent external expert raters, one psychologist (JF) and one emergency physician (WEH), both with extensive simulation experience. Their ratings were recorded using the Team Emergency Assessment Measure (TEAM) checklist [9]. In contrast to many other tools assessing teamwork (for a review, see [41]), TEAM has good psychometric properties such as a high interrater-reliability and high inter-item correlations, has been validated in several translated versions, and used in a variety of simulated and real-life resuscitation scenarios (for a review see [29]). In this study, we employed the German translation of the instrument [29].

The TEAM checklist is a previously published instrument that consists of 11 items assessing teamwork of medical emergency teams on 3 subscales: leadership (2 items), teamwork (7 items), and task management (2 items). All items are rated on a Likert scale ranging from 0 (*never/hardly ever*) to 4 (*always/nearly always*) and are then summarized into a sum score ranging between 0 and 44. In addition, teamwork is rated on a global rating scale (GRS) ranging from 1 (*worst possible performance*) to 10 (*best possible performance*). The GRS was worded as “Please provide an overall evaluation of the non-medical performance of this team.” Because a previous factor analysis [36] identified one underlying component accounting for 81% of the observed variation, with factor loadings > 0.6 and Eigenvalues > 1, it is defensible and common practice to summarize items of the TEAM into a sum score in the range between 0 and 44. The two expert raters each assessed 20% of the videos independently and in duplicate. As inter-rater agreement was good to excellent (intraclass correlation coefficient ICC = 0.87), the remaining videos were rated by a single rater.

We further extracted objective performance measures from the videos. In patients under resuscitation, such measures include early defibrillation and timing and number of doses of adrenaline administered [42], and implementation of extracorporeal circulation (ECC) in hypothermic cardiac arrest [43] and will be referred to as *performance* hereafter.

### Data management and analysis

Questionnaire data were recorded on paper and entered into an excel sheet by an administrative assistant. 20% of the data entered were randomly selected and crosschecked by one of the authors (SCH). Videos were recorded using the AV system permanently installed in the resuscitation bay and stored on a secure network storage drive institutionally approved for storage of health related personal information. Video ratings and the extracted performance data such as time to first defibrillation were directly entered into a spreadsheet by expert raters. All data collected were then imported into an SPSS file. SPSS version 25 (IBM cooperation) was used for data analysis. Data are described using mean or median and standard deviation or range, as appropriate. We calculated Pearson’s correlations between the rater-based measures of teamwork (TEAM sum score and GRS) and participants’ self-evaluation of teamwork. We also correlated the rater-based measures of teamwork with team characteristics such as size, cumulative experience, and age of the team leader. Last, we assessed the relationship between TEAM sum scores or GRS and other behavioural data, such as time to first defibrillation. *P* values of less than 0.05 are considered significant.

## Results

A total of 26 teams participated in the study. Two teams were excluded from the analysis because the videos were not recorded due to a technical failure. One team was excluded because two of its participants did not consent to study participation, and one team because the team contained a physician involved in this study. The analysis was thus based on 22 teams consisting of 115 health care professionals (22 physician team leaders and 93 team members). Participants were on average 36.7 years old and had a mean of 13.1 years’ professional experience, 6.3 years of those in emergency medicine; 77.4% were female (Table 1). Team leaders were on average 36.4 years old and had a mean of 9 years’ professional experience, 3.4 years of those in emergency medicine; 77.3% were female. Each team contained at least one physician (range 1–3, median 1) and two nurses (range 2–4, median 3); no team was smaller than 3 persons (range 3–6, median 4).

**Table 1:** Descriptive statistics

	Mean	Standard deviation	Min	Max
**Participants overall**				
Age [years]	36.7	10.3	20	63
Professional experience [years]	12.7	9.7	0.4	39
Experience emergency medicine [years]	6.3	7.5	0.2	36
Gender	77.4% female			
**Team leader**				
Age [years]	36.4	8.3	29	61
Professional experience [years]	9.0	8.6	2	37
Experience emergency medicine [years]	3.4	5.8	1	27
Gender	77.3% female			
**Scenarios and Performance**				
Total duration [min:sec]	8:15	1:05	5:45	10:15
Time to defibrillation [min:sec]	1:06	0:42	0:09	2:32
Time to adrenaline [min:sec]	3:31	1:34	0:50	6:47^a^
TEAM GRS score [points out of 10]	6.4	1.8	3	10
TEAM sum score [points out of 44]	31.8	5.5	21	42

^a^ Two teams did not administer adrenaline at all.

The objectively observable performance of the teams was highly variable: On average, it took 8 min and 15 s (SD = 1:05 min) before teams called for ECC due to hypothermic arrest (*n* = 9) or the instructor ended the simulation (*n* = 13). Although all teams correctly recognized an arrest requiring early defibrillation, only half (*n* = 11) limited the number of attempted defibrillations, as is best practice in cases of hypothermic arrest. Time to defibrillation (mean = 1:06 min) and time to first adrenaline (mean = 3:31 min) was also highly variable across teams (Table 1). Two teams failed to administer adrenaline at all.

Teamwork quality as reflected by the external observations also varied greatly (Table 1). The TEAM sum score and the GRS were strongly correlated (r_TEAM-GRS_ = 0.92, *p* < 0.001). Those teams with the highest scores on both scales were also the quickest to defibrillate and to provide adrenaline to the patient, providing further evidence for the concurrent validity of the TEAM measurement instrument (Table 2).

**Table 2:** Correlations of external observations, team characteristics and performance

	external observations		team characteristics			performance	
	TEAM GRS Score	TEAM sum score	team size	number of physicians	number of nurses	time to defibrillation	time to adrenaline
**TEAM GRS Score**	1	0.943^**^	0.055	−0.072	0.116	−.463^**^	−.217^*^
**TEAM sum score**	.943^**^	1	0.061	−0.070	0.120	−.451^**^	−.226^*^
**team size**	0.055	0.061	1	.421^**^	.627^**^	−0.048	−0.093
**number of physicians**	−0.072	−0.070	.421^**^	1	−.443^**^	0.193	0.202
**number of nurses**	0.116	0.120	.627^**^	−.443^**^	1	−.214^*^	−.284^**^
**time to defibrillation**	−.463^**^	−.451^**^	−0.048	0.193	−.214^*^	1	.486^**^
**time to adrenaline**	−.217^*^	−.226^*^	− 0.093	0.202	−.284^**^	.486^**^	1

*. Significant on a 0.05 level (two-sided); **. Significant on a 0.01 level (two-sided)

Team members’ self-monitoring judgements of their team’s teamwork were strongly correlated with both of the measures based on external observations (r_TEAM_members-self_ = 0.573, *p* < 0.001, r_GRS_members-self_ = 0.628, *p* < 0.001; Table 3). Interestingly, the same did not apply to the team leaders’ self-monitoring of their team’s teamwork (r_TEAM_leader-self_ = 0.347, *p* = 0.145, r_GRS_leader-self_ = 0.451, *p* = 0.052; Table 4).

**Table 3:** Correlations between self-monitoring, member characteristics and external observations

	Self-monitoring	member characteristics		external observations	
		Age	ER experience	TEAM GRS Score	TEAM sum score
**Self-monitoring**	1	0.095	0.046	0.628^**^	0.573^**^
**Age**	0.095	1	.763^**^	−0.175	−0.140
**ER experience**	0.046	.763^**^	1	−0.162	−0.130
**TEAM GRS Score**	0.628^**^	−0.175	−0.162	1	.944^**^
**TEAM sum score**	0.573^**^	−0.140	−0.130	.944^**^	1

*. Significant on a 0.05 level (two-sided); **. Significant on a 0.01 level (two-sided)

**Table 4:** Correlations between self-monitoring, leader characteristics and external observations

	Self-monitoring	leader characteristics		external observations	
		Age	ER experience	TEAM GRS Score	TEAM sum score
**Self-monitoring**	1	0.651**	0.32	0.451	0.347
**Age**	0.651**	1	0.660**	−0.473*	−0.461*
**ER experience**	0.320	0.660**	1	−0.163	−0.242
**TEAM GRS Score**	0.451	−0.473*	−0.163	1	0.945**
**TEAM sum score**	0.347	−0.461*	−0.242	0.945**	1

*. Significant on a 0.05 level (two-sided); **. Significant on a 0.01 level (two-sided)

Neither team size, nor number of physicians per team, nor cumulative or average experience within a team was associated with the external observations. However, teams led by younger physicians achieved better teamwork scores from external observers than those led by older physicians (r_TEAM_leader-age_ = − 0.461, *p* = 0.047, r_GRS_leader-age_ = − 0.473, *p* = 0.041; Table 4).

## Discussion

The performance of teams in this observational study of leadership in ad hoc teams attending to a simulated cardiac arrest with a rare cause varied greatly, with less than half of all teams making the correct diagnosis and calling for ECC. This variation should not be interpreted as a threat to the quality of actual care, because the whole idea of simulation-based training is to move participants out of their comfort zone to stimulate learning [44]. In fact, the rationale behind training for a hypothermic cardiac arrest was that this algorithm differs in important respects (e.g., dosing of adrenaline; frequency of defibrillation; early initiation of ECC) from standard advanced cardiac life support [43], and hypothermic arrests occur regularly (although infrequently) in our catchment area [37, 45, 46]. The scenario thus tested the team leader’s ability to lead the team through an algorithm that differs substantially from the much more common standard advanced cardiac life support [47], thus offering a potent opportunity for learning.

This heterogeneity in objective performance across teams is reflected in both, the external observation measures of video recordings and the teams’ self-monitoring, a finding that directly answers our second research question. The fact that objective performance measures of resuscitations correlate with scores on the TEAM checklist further validates this instrument for the assessment of resuscitation teams, a finding in line with previous research [9, 36]. It is interesting to note that the TEAM instrument assesses quality of teamwork, not quality of a resuscitation per se, but it seems that good teamwork is a prerequisite for good resuscitation [9, 36]. This finding, which we replicate, is reassuring, because arguably, the sole purpose of attending to patients in cardiac arrest with a team is to provide good resuscitation.

With regard to the first research question, we found moderately high agreement between the external observations and team members’ self-monitoring of their teams’ teamwork. These findings are well in line with the literature on self-monitoring of one’s individual performance on a moment-by-moment basis [31, 35, 39, 40, 48]. Our study extends this conception in two important ways. First, it indicates that the ability to accurately self-evaluate applies to both concurrent monitoring during an event and the time shortly after this event has concluded. Second, it suggests that individuals are capable of monitoring not only their own performance but also that of a team to which they belong. Interestingly, this finding does not hold for team leaders evaluating their team’s teamwork.

There are two possible explanations for the latter finding. One is that evaluating a team’s teamwork as a team member is a substantially different task than evaluating this team’s teamwork as the team leader. Every physician team leader carries the responsibility for their team’s teamwork, because arguably a key purpose of instituting a physician team leader is to ensure good teamwork in ad-hoc teams. Given the complexity of leadership [3, 10, 12, 13, 49], particularly in highly dynamic environments such as emergency rooms, leaders may be operating at their full capacity and lack the necessary mental resources for additional meta-cognitive tasks such as self-monitoring. The diffusion of responsibility observable even in small groups [50, 51] may also lead to this diffused responsibility being burdened on the team leader when teams institute such a function explicitly, resulting in further leader overload. It remains an open question whether this phenomenon also affects teams led by experienced non-physicians such as advanced practice nurses. Such nurses may be less tempted than physicians to focus on things other than teamwork in their role as team leaders. Also, nurses in emergency medicine often have considerably longer experience in the field than the physicians they work with. In this study, for example, team members (i.e., mostly nurses) had about twice as much experience in emergency medicine as physician team leaders. Although we cannot exclude that professional experience plays a role in the ability to self-monitor oneself, the literature on self-monitoring suggests otherwise (e.g. [39, 40]).

Another reason for that we did not observe adequate self-monitoring in team leaders may simply be the small sample size of just 22 team leaders. This sample may be too small to achieve significance when testing the likelihood of a correlation occurring by chance. However, we did find significant correlations between team performance and leaders’ age at this sample size. Sample size remains a notorious challenge in small group research [2], and this study is no exception.

Other limitations of this study result from its observational design, which was chosen to mimic real-world circumstances as closely as possible, but which rendered it impossible to control for and/or manipulate variables that may affect performance, such as team size, structure, or heterogeneity [52]. This limitation is, at the same time, one of the key strengths of the study: real-world ad hoc teams are also diverse with respect to all these variables and change on a frequent basis. The only such variable we found to affect teamwork was the age of the team leader. We can only speculate that this is because younger team leaders may have more recently received training on resuscitation guidelines, but this finding requires closer investigation. In addition, other factors that may affect teamwork such as institutional culture and environment were not varied between teams in this study, potentially limiting generalizability of our findings to environments substantially different from the one investigated here. Last, the simulation is limited by the fact that a hint was necessary to tell participants that their hands went cold during chest compression, because the simulator used cannot be programmed to feel cold.

## Conclusion

Team members seem to have better insight into their teams’ teamwork than team leaders, as indicated by the moderately high correlation of their self-monitoring ratings with external ratings of their teamwork. As a practical consequence, the decision to debrief after a resuscitation and the debriefing itself should be informed by team members, not just leaders. External ratings of teamwork as recorded with the TEAM instrument were substantially correlated to objective measures of team performance, a finding that further adds to the validity of the TEAM assessment instrument.

## Data Availability

The datasets used and analysed during the current study are available from the corresponding author on reasonable request for researchers eligible to work with human data according to Swiss legislation. Eligibility will be determined by the ethics committee of the Canton Bern.

## Abbreviations

ISS: Injury Severity Score
ECC: Extracorporeal circulation
TEAM: Team Emergency Assessment Measure
GRS: Global rating scale
ICC: Intraclass correlation coefficient
ANP: Advanced nurse practitioners
CRM: Crew resource management
